# SMBE Secretary's Report 2025

**DOI:** 10.1093/molbev/msaf280

**Published:** 2025-12-08

**Authors:** Emmanuelle Lerat

**Affiliations:** Universite Claude Bernard Lyon 1, LBBE, UMR 5558, CNRS, VAS, Villeurbanne, F-69622, France

**Council Meetings**

## I. Call to Order

President Ziheng Yang called the council meeting to order at 8:45 AM China Standard Time on July 20, 2025. In the presence of President Ziheng Yang, Past-President Stephen Wright, President-Elect Juliette de Meaux, Councilors Juliana Vianna and Aya Takahashi, Secretary Emmanuelle Lerat, MBE Editor-in-Chief Claudia Russo (*ex officio*), and GBE Editor-in-Chief Maud Tenaillon (*ex officio*).

*Non-council Members in Attendance:* Kimi Zeng (Head of China Journals OUP), Ravinder Kanda and Epifanía Arango-Isaza (IDEA), Serena Dai and Xuming Zhou (LOC SMBE2025).

*Council Members in virtual attendance:* Councilors Christian Landry, Rebecca Zufall, Elena Gomez-Diaz, and Melissa Wilson, Treasurer Aline Muyle, MBE Editor-in-Chief Brandon Gaut (*ex officio*), and GBE Editor-in-Chief Laura A. Katz (*ex officio*).

*Non-council Members in virtual attendance:* Chris Lapine (KGL), Andy Seagram (Publisher OUP, MBE & GBE) and Tom Gilbert (LOC SMBE2026).

## II. Treasurer's Report

Treasurer Aline Muyle presented the annual Treasurer's report, which included details on journal and meeting revenue and expenses, as well as a projection for the upcoming year. While the society is financially healthy globally, its net assets have been decreasing since 2022. The 2025 projection also shows a deficit, though it should not be too high. Efforts have been made to secure investments with competitive rates. The council discussed several priorities to best serve our membership moving forward. Different ideas were proposed to increase income and decrease expenses, including rethinking the organization of the annual meetings, which are the biggest expense. Full details can be found in the complete Treasurer's Report.

## III. Secretary's Report

Secretary Emmanuelle Lerat gave a brief review of the Council's actions over the preceding year. Between January 1 and June 2025, the Council voted on six motions. The Council approved the following: (1) regional and satellite meeting choices, (2) the new KGL contract, (3) the renewal of Paula Brantner's position as Safe SMBE officer for another year, (4) the 2026 article processing charges (APCs), (5) the report regarding the code of conduct violation during SMBE2024, and (6) the faculty awards. Several other motions were discussed and put to a vote between July and December 2024: (1) Approval of the SMBE2027 Quebec proposal without a call for submissions; (2) Evaluation of potential support for the Mastodon ecoevol.social server; (3) Inquiry from the International Society for Biomolecular Archaeology; (4) Appointment of a new EiC for GBE; (5) Appointment of new GBE associate editors; (6) Approval of SMBE2027 in Quebec City; (7) AI licensing opportunities for SMBE; (8) A funding proposal for editors and council members to attend SMBE2025; and (9) The opening of a social media editor position. Full details can be found in the complete Secretary's report.

## IV. Election of SMBE Officers

Harmit Malik chaired the nomination committee, which proposed candidates for the positions of President-Elect, Secretary, Treasurer, and two Councilors. The committee members were Xuming Zhou, Mary O’Connell, Nicolas Galtier, and Deepa Agashe. Emmanuelle Lerat (SMBE Secretary) was the *ex officio* member, as per the bylaws. In early 2025, the society's membership was informed by email of the committee's composition and asked to nominate candidates for the positions. The council reminds the nominating committee every year to prioritize gender and geographic representation among the candidates but allows flexibility in how the committee achieves this.

The final nominees selected were Josefa Gonzalez and Matthew Hahn for President-Elect; Wenfeng Qian and Rebekah Rogers for Secretary; Amandine Cornille and JJ Emerson for Treasurer; and Qiaomei Fu, Eric Gaucher, Ravinder Kanda, and Li Zhao for Councilors. Ballot details were issued to society members online through KGL. A total of 660 members voted in this election out of 2,812 invited members. This corresponds to a 23.75% participation rate, higher than last year's. Matthew Hahn was elected President-Elect, Rebekah Rogers was elected Secretary, JJ Emerson was elected Treasurer, and Ravinder Kanda and Qiaomei Fu were elected Councilors.

## V. Editors’/OUP/AP Reports

The Editors-in-Chiefs of MBE and GBE presented reports on the current state of their respective journals. See the editors’ reports for full details. Pedro Andrade was appointed Social Media Editor to handle social media for MBE, GBE, and SMBE and to write highlights for both journals. In 2024, the two journals published a series of 11 monthly perspectives, each accompanied by a virtual issue. This series highlighted the 40th anniversary of the SMBE journals. GBE's submission numbers have returned to pre-pandemic levels. Its impact factor (IF) is stable, and the journal ranks in the top 34% in the evolutionary biology domain. Efforts are needed to maintain the number of submissions, increase citations, and maintain commitment to inclusive publishing. The MBE IF dropped in 2024, but this has no influence on the strong 5-year IF (11.9).

## VI. Other Activities

### Future Annual Meetings

SMBE2026 will take place in Copenhagen, Denmark. SMBE2027 will take place in Quebec City, Canada. The decision was made to make the two meetings hybrid to allow for better accessibility. Additionally, it was decided that no annual meeting will be held in 2028 and it will be replaced by several regional meetings. The next annual meeting will be held in 2029, alternating with extended regional meetings the following year. At that time, a survey will be conducted to evaluate the continuation of this shift.

### IDEA Task Force, Proposals

The call for IDEA proposals resulted in the funding of one proposal: Building ScienceWise (proposed by Rori Rohlfs (Oregon University) and Emilia Huerta-Sanchez (Brown University). An outreach project was funded: Regional Science Olympiad of Evolutionary Biology (ORBE2024) (proposed by Claudia Russo (Federal University of Rio de Janeiro)). Two non-parachute-science projects were funded: 1. Bridging the Divide: Empowering African Researchers in Molecular Biology and Bioinformatics (proposed by Boris Olou and collaborators (University of Parakou, Benin); 2. Developing solutions to teach molecular biology and evolution within West African countries with limited laboratory infrastructures: the use of miniaturized laboratory equipment in Guinea-Bissau (proposed by Stefan Prost (University of Oulu) and Aissa Regalla (IBAP, Guinea Bissau)). The next IDEA priorities are launching the 2026 call for proposals to support IDEA-focused initiatives in SMBE and the broader MBE community; expanding the IDEA committee and its subcommittee volunteer base, especially in parachute science and outreach; and broadening SMBE membership in underrepresented regions and communities. The focus will be on long-term inclusion.

### Carer Awards

Twenty two people were supported by this award to attend the SMBE2025 meeting in Beijing, China.

### Early Career Award

This award is given to outstanding members of the SMBE community who are in the early stages of an independent research career. The 2025 recipient was Alison Feder from the University of Washington (USA).

### Mid-Career Award

This award is intended for outstanding members of the SMBE community who are in the midst of their research careers. The 2025 recipient was Amanda Larracuente of the University of Rochester (USA).

### Lifetime Achievement Award

This award is intended for outstanding senior members of the SMBE community. The 2025 recipient was Chung-I Wu from the University of Chicago (USA).

### Graduate Student Excellence Awards

This Symposium provides a forum for young investigators to showcase their exemplary research and continues to be a highlight of the SMBE annual meeting. We celebrate all of the candidates in this symposium. The awardees were:

Huangyi He (Zhejiang University, China)

Alba Nieto Heredia (Université PSL—MNHN, France)

Philipp Hummer (Vienna Graduate School of Population Genetics, Austria)

Kaiyuan Li (UC Berkley, USA)

Gemma Martinez-Redondo (Institute of Evolutionary Biology, Spain)

Aidan Short (University of South Carolina, USA)

Dylan Sosa (University of Chicago, USA)

Hannah Verdonk (Temple University, USA)

### Best Graduate Student Paper Award

These awards provide recognition for outstanding student papers in both SMBE journals. For MBE, the committee selected one winner and one honorable mention.

*Winner:*

Julien Joseph. “Increased Positive Selection in Highly Recombining Genes Does not Necessarily Reflect an Evolutionary Advantage of Recombination”. https://academic.oup.com/mbe/article/41/6/msae107/7686984

*Honorable Mention*:

William Henriques. “The Diverse Evolutionary Histories of Domesticated Metaviral Capsid Genes in Mammals”.

https://academic.oup.com/mbe/article/41/4/msae061/7632644

For GBE, the committee selected one winner and one honorable mention.

*Winner*:

Laura C Fricke. “Identification of Parthenogenesis-Inducing Effector Proteins in Wolbachia”, https://academic.oup.com/gbe/article/16/4/evae036/7635173

Honorable mention to Jiachen Li. “Where Are the Formerly Y-linked Genes in the Ryukyu Spiny Rat that has Lost its Y Chromosome?” https://academic.oup.com/gbe/article/16/3/evae046/7628192.

### SMBE Young Investigator Travel Award

These awards were granted to 14 graduate students or postdoctoral researchers to attend the SMBE2025 meeting in Beijing, China.

## Business Meeting

The business meeting took place on July 22, 2025, during the SMBE conference in Beijing, China. The President recognized the efforts of the local organizing committee for the first meeting in China. He provided a financial overview of the society and detailed the various awards presented during the event. Additionally, the President offered insights into membership categories, updates, and the benefits of membership. He emphasized the advantages of publishing in the SMBE journals, especially for society members. The election results were announced, along with the locations of SMBE2026 and SMBE2027, and the new rotation of annual and extended regional meetings, which will begin in 2028. The President also outlined the upcoming regional and satellite meetings and highlighted the work of the IDEA task force.

